# Evidence‐Based Medicine Within Surgical Practice and Training: A Scoping Review

**DOI:** 10.1002/wjs.12479

**Published:** 2025-02-04

**Authors:** E. Tokidis, G. Perin, P. Vivekananda‐Schmidt, S. P. Balasubramanian

**Affiliations:** ^1^ Division of Clinical Medicine School of Medicine and Population Health University of Sheffield Sheffield UK; ^2^ Department of General Surgery Doncaster and Bassetlaw NHS Foundation Trust Doncaster UK; ^3^ Department of Colorectal Surgery Leeds Teaching Hospitals NHS Trust Leeds UK; ^4^ Department of General Surgery Sheffield Teaching Hospitals NHS Foundation Trust Sheffield UK

**Keywords:** evidence‐based medicine, post‐graduate education, resident education, surgery, surgical education

## Abstract

**Background:**

Evidence‐based medicine (EBM) is a fundamental element of modern surgical practice. However, its integration into surgical training remains challenging. Through a scoping review, this study mapped existing evidence on the practice of EBM in surgery, focusing on knowledge, attitudes, barriers, and curricula.

**Methods:**

The literature was systematically searched through 3 databases and 2 registers. Fifty articles published over a 22‐year period (2001–2023) were identified based on predefined eligibility criteria. These were reviewed using Arksey and O'Malley's methodological framework. The review was checked against the PRISMA 2018 checklist for scoping reviews.

**Results:**

There is evidence of awareness and appreciation of EBM principles among surgeons and surgical trainees. However, the understanding of EBM terminology is varied. Attitudes toward EBM were predominantly positive yet reliance on clinical experience over evidence‐based guidelines was noted. Key barriers to EBM practice included time constraints, lack of structured training, and methodological challenges in surgical research. The review highlighted the lack of validated competency assessment tools and the need for structured EBM curricula. Various educational strategies, such as journal clubs and courses, were found to improve EBM knowledge, albeit with limited evidence on long‐term practice change.

**Conclusion:**

This scoping review underscores the need for a more detailed understanding of stakeholder views of EBM in surgical practice, the development, implementation, and assessment of educational interventions in this field, and tailored strategies to assess EBM competency in surgery.

## Graphical abstract

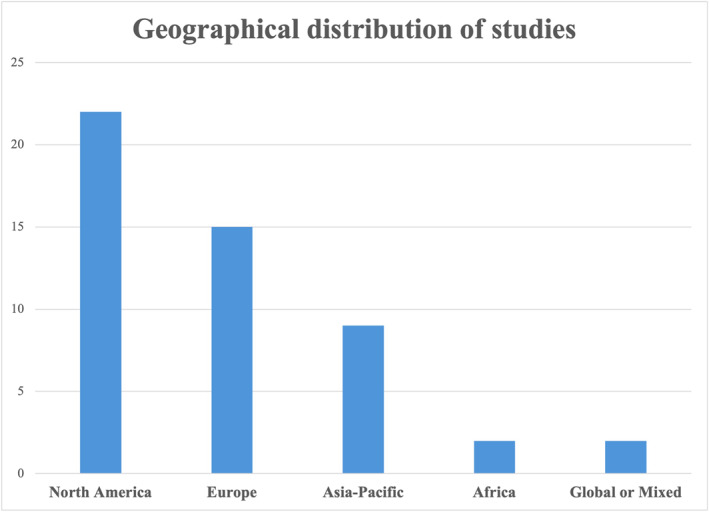

## 1 Introduction

Evidence‐based medicine (EBM) defined as ‘the conscientious, explicit, and judicious use of current best evidence in making decisions about the care of the individual patient [1]’ constitutes an inherent part of modern healthcare [2]. In 2018, Albarqouni et al. [3] published a Delphi consensus on EBM competency which describes the set of competencies to be achieved by healthcare professionals. A year later, Thoma et al. [4], followed with ‘evidence‐based Surgery’ as an aid to surgical practice. However, integrating evidence‐based medicine competency training into surgical postgraduate curricula is slow because of factors such as limited time within already demanding training schedules, insufficient faculty expertise in EBM, and lack of structured and standardized training frameworks [5].

Furthermore, surgical training has traditionally followed an apprenticeship model [6]. This hinders the integration of evidence‐based surgery due to several factors including lack of integration of EBM principles into training curricula, limited availability of educational resources, time constraints faced by trainees and trainers, and the perception that obtaining EBM competency is of lower priority compared to technical skills [7]. Furthermore, the complexity of surgical practice leads to methodological challenges in surgical studies [8]. At present, structured teaching, systematic training, and validated assessment tools for evaluating EBM competency remain inadequately developed in surgical education.

Key to integrating EBM in surgery is a comprehensive understanding of EBM related knowledge, attitudes, and educational needs within surgical practice alongside an examination of teaching methodologies and assessment tools [9] available to surgeons. This scoping review aims to identify the relevant literature on the topic and provide an overview of published evidence on the practice of EBM in surgery, with a particular focus on attitudes, training, and curriculum in EBM, and assessment of EBM competencies.

## 2 Methods

This study is registered on the open science framework (OSF) (doi: 10.17605/osf.io/6vzm3). The five stage framework as described by Arskey and O'Malley for performing a scoping review [10] was followed. The scoping review has been checked against the 2018 PRISMA checklist for scoping reviews.

### 2.1 Framework Stage 1: Identification of the Research Question

We aimed to address the following research questions:

1. What is the knowledge and understanding of EBM as defined by Sacket and outlined earlier in the introduction amongst surgeons and surgical trainees?
2. What are the attitudes of surgeons and surgical trainees toward EBM and their perceived barriers in the practice of EBM?
3. How are surgeons trained and assessed in EBM?

### 2.2 Framework Stage 2: Identification of Relevant Studies

The terms *“evidence‐based medicine, evidence‐based surgery, evidence‐based practice, surgery, surgical practice, training, teaching, perception, awareness, attitude, need, curriculum, assessment, utility, tool, critical appraisal, journal club”* were combined and searched in PubMed, Ovid Medline, Embase, ERIC, and White Rose Research Online (research repository of University of Sheffield, Leeds and York; recommended as a search database by the university's doctoral development program) on 20th January 2024. A further search of citations from two related systematic reviews [9, 11] as well as hand search via ‘PubMed similar articles’ function was conducted (See Supporting Information S1 for details).

### 2.3 Framework Stage 3: Study Selection

All published original or secondary research manuscripts in the English language; on knowledge, understanding, attitudes, barriers, teaching, training, or assessment of EBM in surgery were included.

### 2.4 Framework Stage 4: Charting the Data

All articles were downloaded to Rayyan software and after removing duplicate articles, the titles and abstracts were screened by two investigators (E.T. and G.P.) to identify relevant articles. Manuscripts not addressing EBM in surgery, editorials, and commentary papers and nonEnglish language papers were excluded. One investigator (E.T.) reviewed full‐text of the selected articles to ensure eligibility and a sample of the full‐texts was reviewed by another investigator (G.P.) for additional validation.

Data from the included manuscripts were collected on an excel spreadsheet. Publication details, study characteristics, and details on specialty and participants were included. Additionally, full‐texts were reviewed for the concepts related to this study's research questions as described at stage one. The data related to the concepts were organized based on the key findings of each study.

Reflexivity [12], understood as the potential influence of researchers' perspectives on the scoping process, was acknowledged. The lead author (E.T.), is a surgical trainee and postgraduate researcher. He led the review design and analysis. The second author (G.P.) is also a surgical trainee with significant experience in surgical research and running journal clubs. The review process was primarily conducted by these two authors (E.T., G.P.), with regular oversight from senior surgical (S.P.B.) and medical education (P.V.‐S.) team members who provided feedback and ensured methodological rigor.

## 3 Results

### 3.1 Framework Stage 5: Collating, Summarizing and Reporting

**FIGURE 1:**
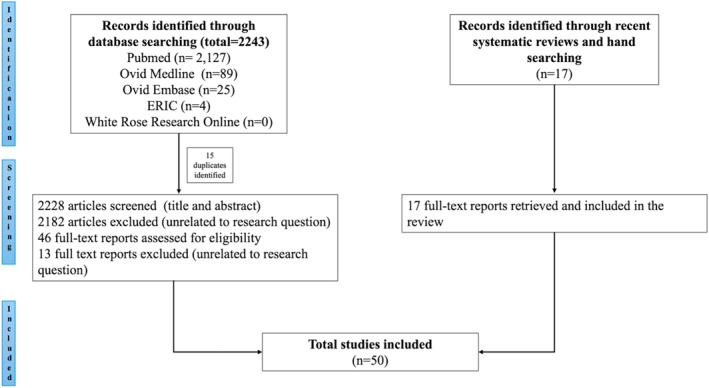
PRISMA flowchart.

**FIGURE 2:**
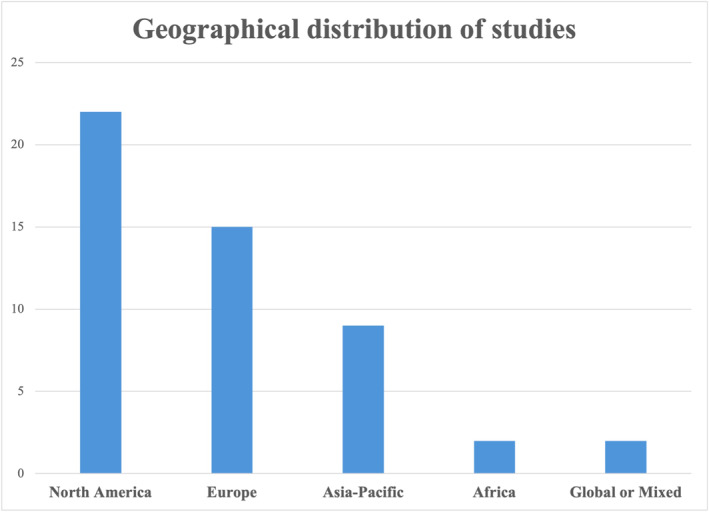
Geographical distribution of included studies.

A total of 50 articles published across a period of 22 years (2001–2023) were included (Figure 1). The included studies and detailed study characteristics are listed in Table 1. The studies identified were primarily conducted in the United States of America, Canada, and Netherlands (Figure 2) and conducted across most surgical specialties (Table 1).

**TABLE 1:** Study characteristics.

First author	Study date	Country	Journal	IF	SJR	Design	Specialty	Theme
Siegrist and Giger [13]	2006	Switzerland	Swiss Med Wkly	2.9	0.575	Survey (web‐based)	Multiple specialties	Teaching and Training/Curriculum
Rademaker et al. [14]	2019	Netherlands	PLoS One	3.7	0.885	Survey (web‐based)	ENT	Attitudes/Barriers
Stapleton, Cuncins‐Hearn, and Pinnock [15]	2001	Australia	ANZ J Surg	1.7	0.406	Survey (paper based)	Urology	Knowledge/Understanding/Attitudes
Knops et al. [16]	2009	Netherlands	World J Surg	2.6	0.902	Survey study and quiz	General Surgery	Knowledge/Understanding/Attitudes
Scales [17]	2008	United States	J Urol	6.6	1.945	Survey study (web‐based questionnaire)	Urology	Perceptions/Attitudes/Barriers
Bhandari et al. [7]	2003	Canada	Acad Med	7.4	1.579	Focus groups and semi‐structured interviews	Multiple surgical specialties	Perception/Attitude/Barriers
Hajebrahimi et al. [18]	2014	Iran	Urol J	1.5	0.396	Survey (web‐based)	Urology	Knowledge/Perception/Attitudes/Barriers
Lugano et al. [19]	2020	Global (mainly European)	Knee Surg Sports Traumatol Arthrosc	9.8	3.292	Survey (web‐based)	Orthopedics	Knowledge/Barriers
Schnitzbauer et al. [20]	2014	Germany/UK	Eur Surg Res	1.6	0.344	Survey (web‐based)	General Surgery	Attitudes/Barriers
S. Kitto et al. [21]	2011	Australia	J Eval Clin Pract	2.4	0.782	Semi‐structured interviews	Multiple surgical specialties	Knowledge/Understanding/Attitudes
Sur et al. [22]	2006	USA	J Urol	6.1	1.945	Survey (web‐based)	Urology	Knowledge/Understanding/Attitudes
Kumar et al. [23]	2011	India	Ann R Coll Surg Engl	1.4	0.335	Survey (web‐based)	Orthopedics	Knowledge/Understanding/Attitudes/Barriers
Mittal and Perakath [24]	2010	India	J Surg Educ	2.9	0.935	Survey (web‐based)	Multiple surgical specialties	Attitudes/Barriers
Santori et al. [25]	2011	Italy	Transplant Proc	0.9	0.316	Cross‐sectional study	General Surgery	Teaching and Training/Assessment/Curriculum
Wolf et al. [26]	2009	USA	Orthopedics	1.1	0.523	Cross‐sectional study	Orthopedics	Knowledge/Teaching and Training
Mildon et al. [27]	2001	Canada	Can J Ophthalmol	4.2	0.589	Survey	Ophthalmology	Knowledge/Understanding/Attitudes//Barriers
Johnson et al. [28]	2014	USA	J Surg Educ	2.9	0.935	Cross‐sectional study	Surgery unspecified	Knowledge
Poolman et al. [29]	2007	Netherlands	J Bone Joint Surg Am	3.3	1.996	Survey (web‐based)	Orthopedics	Knowledge/Attitudes/Needs/Barriers
Amin, Saunders, and Fenton [30]	2007	Ireland	Clin Otolaryngol	2.1	0.782	Survey (paper based)	ENT	Knowledge/Attitudes
Sprague et al. [31]	2012	Canada	Can J Surg	2.5	0.525	Cross‐sectional study	Multiple surgical	Teaching and Training/Assessment
Ahmadi et al. [32]	2013	Canada	Can J Surg	2.5	0.525	Survey (web‐based)	General Surgery	Teaching and Training/Curriculum
S. Kitto et al. [33]	2007	Australia	ANZ J Surg	1.7	0.406	Pilot survey (web‐based)	Rural Surgery	Understanding/Attitudes
S. C. Kitto et al. [34]	2011	Australia	J Eval Clin Pract	2.4	0.782	Survey (web‐based)	Rural Surgery	Attitudes/Barriers
Haines and Nicholas [35]	2003	USA	J Am Coll Surg	5.2	1.534	Programme development	Neurosurgery	Teaching and Training/Curriculum
Komenaka et al. [36]	2015	USA	J Surg Educ	2.9	0.935	Prospective cohort study	General Surgery (breast surgery rotation)	Teaching and Training/Assessment/Curriculum
Toedter, Thompson, and Rohatgi [37]	2004	USA	J Am Coll Surg	5.2	1.534	Programme development	Surgery unspecified	Teaching and Training/Assessment/Curriculum
Tam et al. [38]	2011	Taiwan	J Eval Clin Pract	2.4	0.782	Survey study	General Surgery	Curriculum
Weller et al. [39]	2023	Netherlands	BMJ Open	2.9	1.059	Semi‐structured interviews	Medicine and Surgery	Attitudes
Ubbink et al. [40]	2016	Netherlands	World J Surg	2.6	0.902	Cross‐sectional study	Multiple specialties	Teaching and Training/Assessment
Temple and Ross [41]	2011	Canada	J Surg Educ	2.9	0.935	Cross‐sectional study	Plastic Surgery	Teaching and Training/Assessment/Curriculum
Fischer et al. [42]	2009	Germany	J Surg Educ	2.9	0.935	Survey (web‐based)	Surgery unspecified	Teaching and Training
Hong and Chen [43]	2019	China	Int J Environ Res Public Health	3.2	0.828	Survey (web‐based)	Medicine and Surgery	Knowledge/Perceptions/Attitudes/Barriers
Goldhahn et al. [44]	2007	UK	BMC Med Educ	3.6	0.914	Survey (web‐based)	Medicine and Surgery	Knowledge/Perceptions/Attitudes
Trickey et al. [45]	2005	Multiple	J Grad Med Educ	2.7	0.988	Cross‐sectional study	General Surgery	Teaching and Training/Assessment/Curriculum
MacRae et al. [46]	2014	USA	Am J Surg	0.5	0.502	Cross‐sectional study	General Surgery	Assessment
Ubbink et al. [47]	2023	Netherlands	Cureus	1.2	n/a	Cross‐sectional study	General Surgery	Teaching and Training/Assessment
Duong et al. [48]	2022	USA	J Surg Educ	2.9	0.935	Survey (web‐based)	ENT	Teaching and Training/Curriculum
Luc et al. [49]	2022	USA	Ann Thorac Surg	4.6	1.27	Survey (web‐based) and cross‐sectional study for pre–post test scores	Cardiothoracic Surgery	Curriculum
Luc et al. [50]	2017	USA	Ann Thorac Surg	4.6	1.27	Survey (web‐based) and cross‐sectional study for pre–post test scores	Cardiothoracic Surgery	Curriculum
Williams et al. [51]	2022	USA/Canada	J Surg Educ	2.9	0.935	Cross‐sectional survey	General Surgery	Teaching and Training/Curriculum
Hryciw Knox, and Arneja [52]	2019	USA	Plast Surg(Oakv)	0.947	n/a	Survey (web‐based)	Plastic Surgery	Attitudes/Barriers/Teaching and Training/Curriculum
Mullen and Sabri [53]	2017	USA/Canada	Can J Ophthalmol	4.2	0.589	Survey (web‐based)	Ophthalmology	Teaching and Training/Curriculum
Teunis et al. [54]	2016	Canada	Clin Orthop Relat Res	4.2	1.18	Survey (web‐based)	Orthopedics	Attitudes
Herur et al. [55]	2016	India	Ann Med Health Sci Res	n/a	n/a	Cross‐sectional study	Medicine and Surgery	Teaching and Training/Assessment/Curriculum
Ibrahim et al. [56]	2016	Nigeria	Niger J Surg	n/a	n/a	Survey (web‐based)	General Surgery	Teaching and Training/Curriculum
Lao, Puligandla, and Baird [57]	2014	Canada	J Pediatr Surg	2.4	0.87	Cross‐sectional study	Pediatric Surgery	Teaching and Training/Curriculum
Roths [58]	2014	Canada	J Surg Educ	2.9	0.93	Survey (web‐based)	Urology	Knowledge/Attitudes/Barriers
Dunning [59]	2011	UK	ANZ J Surg	1.7	0.40	Description of the design of an evidence‐based medicine journal club and curriculum	Cardiothoracic Surgery	Curriculum
Toouli and Stanton [60]	2010	Australia	Interact Cardiovasc Thorac Surg	1.7	0.53	Descriptive study	Surgery unspecified	Teaching and Training
Fritsche [61]	2004	Germany	BMJ	107.7	2.867	Cross‐sectional study	Medicine and Surgery	Teaching and Training/Assessment

Most studies identified in this scoping review were mixed‐method surveys (*n* = 29), followed by studies of educational interventions (*n* = 18), and qualitative studies (*n* = 3). The response rate for the survey studies varied from 8.8% to 100%. There is significant overlap in the themes explored by the surveys reported by the different studies. These included knowledge and understanding of EBM, attitudes toward EBM, perceived barriers to the practice of EBM in surgery, training and assessment of competency in EBM, and EBM curricula in surgery.

#### 3.1.1 Knowledge and Understanding of EBM

Thirteen studies investigated surgeons' and surgical trainees' knowledge and understanding of EBM. Eight studies [16, 18, 19, 22, 29, 30, 43, 58] assessed the knowledge of participants by reviewing their familiarity with EBM terminology chosen by the authors of these studies. The knowledge of the terminology varied significantly amongst surveys. In a study of rural surgeons [21], EBM was perceived as ‘the conscientious, explicit, and judicious use of current best evidence in making decisions about the care of the individual patient’ [1]. Overall, surgeons and trainees demonstrated familiarity with EBM. However, only 25%–75% of respondents were confident about the terminology related to EBM.

Only one study [62] used a validated measure of knowledge (numeracy), utilizing the Schwartz–Woloshin tool [63]. Kumar et al. [23] and Poolman et al. [29] noted a positive association between respondents with a higher academic degree such as PhD and knowledge and understanding of EBM concepts. Conversely, Lugano et al. [19] did not identify this association in their survey. Awareness and usage of EBM resources were explored by 7 studies [18, 22, 23, 27, 29, 43, 58]. The resources included in the surveys were journals, specialty specific guidelines, and major databases such as PubMed or Cochrane. Guidelines and major databases were the main points of reference for most respondents.

#### 3.1.2 Attitudes Toward EBM

Twenty studies examined surgeons' and surgical trainees' attitudes toward EBM; 18 were mixed‐method surveys. S. Kitto et al. [21] and Weller et al. [39] chose a solely qualitative approach (interviews) to capture attitudes. Certain studies [14, 16, 24, 29, 30] primarily used the McColl questionnaire [64], and others used validated [18, 34] tools for assessing attitudes.

Overall, these studies show positive attitudes of surgeons and surgical trainees regardless of their surgical subspecialty. Rademaker et al. [14] and Kumar et al. [23] reviewed whether holding a higher academic degree (MD or PhD) influences attitudes. The latter found that those who have a higher degree of proficiency are less inclined to change their practice in response to new evidence, whereas the former found no differences in attitudes. S. Kitto et al. [21] observed specific surgical practice traits that resembled ‘entrepreneurs’ who relied on experience over science and EBM, ‘scientists’ who were research and EBM focused and ‘clinicians’ who followed a more ‘holistic approach’.

Weller et al. [39] concluded that specialists primarily rely on their general knowledge and experience, rather than actively using information sources (‘scientific publications’, ‘guidelines or protocols’, and ‘presentations and meetings’). Distinct strategies were employed for complex clinical queries: consulting a colleague, actively searching the literature, and delegating the literature search [65]. Teunis et al. [54] in their survey observed that variables such as confidence bias, trust in orthopedic evidence base, and statistical understanding are linked to inadequate detection of uncertainty.

#### 3.1.3 Barriers to Practice of EBM in Surgery

Barriers to EBM implementation were explored by 10 studies. The BARRIERS scale [66] was used in two of these [14, 16]. The rest of the studies explored barriers based on survey findings on attitudes toward EBM or by analysis of qualitative data [7, 34]. Time [7, 14, 18, 19, 23, 24] and lack of EBM training [7, 17, 20] were identified by the majority of the included studies as major barriers for EBM implementation in practice. Hierarchical structures as a barrier to EBM were explored by Bhandari et al. [7], Hajebrahimi et al. [18], and Schnitzbauer et al. [20]. Moreover, institutional barriers [7, 18, 20], methodological inadequacies [16, 17, 20], and access to evidence resources or evidence summary documents were common themes identified as barriers in the included studies. These are explained further in the discussion section.

#### 3.1.4 Training in EBM and Assessment of EBM Competency

Table 2 outlines various studies on training in evidence‐based medicine (EBM) and the assessment of EBM competency in surgery. It categorizes studies based on their use of validated or nonvalidated assessment methods. Studies using validated tools like the Fresno tool [31, 55] and Berlin questionnaire [40, 61] demonstrated statistically significant improvements in EBM knowledge and critical appraisal skills among surgical trainees and residents. Studies using nonvalidated assessments [25, 26, 36, 42] also showed positive outcomes such as improved test scores. However, the data from studies lacking standardized measures provided less robust comparisons. Overall, the studies evaluated emphasize the effectiveness of structured EBM training in enhancing knowledge and application among surgical trainees.

**TABLE 2:** Details of studies on training in EBM and the assessment of EBM competency in surgery.

	Study	Method of training	Assessment	Learners	Key findings
Studies using validated assessment methods	Sprague et al. [31]	EBM course	Fresno tool	Surgical trainees	Participants showed a statistically significant improvement in EBM knowledge post‐course, measured by the Fresno tool. The course was effective in transferring knowledge about EBM principles and application.
	Herur et al. [55]	Single session EBM journal club	Fresno tool	Surgical trainees	Journal club session led to a significant increase in the mean overall EBM scores and individual step scores in the EBM process, indicating enhanced understanding and application of EBM principles among participants.
	Ubbink et al. [40]	2‐day critical appraisal course	Berlin questionnaire	Surgical residents	2‐day critical appraisal course resulted in significant improvement in participants' post‐test results using the Berlin questionnaire, demonstrating effectiveness in teaching critical appraisal skills.
	Fritsche et al. [61]	3‐day critical appraisal course	Berlin questionnaire	Surgical residents	A 3‐day critical appraisal course showed statistically significant improvement in post‐test results, confirming the course's effectiveness in enhancing EBM knowledge and skills.
	MacRae et al. [46]	Exam test assessing EBM competency	MacRae tool	General surgery trainees	General surgery trainees with a strong background in critical appraisal performed significantly better in the exam test designed to assess EBM competency, highlighting the importance of prior knowledge and training in critical appraisal.
Studies using nonvalidated assessment methods	Santori et al. [25]	10 sessions on EBM	Session‐specific MCQ	Surgical trainees	Throughout the curriculum, session‐specific MCQs led to statistically significant improvement in post‐test results, showing that frequent assessments can enhance learning and retention of EBM knowledge.
	Wolf et al. [26]	Training to grade level of evidence	Grading levels of evidence in 10 articles	Orthopedic residents	Orthopedic residents who participated in the study showed significant improvement in their ability to grade levels of evidence in 10 articles post‐test, demonstrating the effectiveness of the training in improving critical appraisal skills.
	Komenaka et al. [36]	Training to critical appraise articles	Nonvalidated written exam (based on ABSITE)	General surgery residents	Nonvalidated written examination correlated with ABSITE scores showed that the EBM curriculum positively impacted breast cancer knowledge and residents' satisfaction, suggesting a beneficial effect on their overall learning and performance.
	Fischer et al. [42]	Survey evaluating postgraduate surgical investigator course	No assessment tool	Postgraduate surgical investigators	Survey evaluating a postgraduate surgical investigator course indicated high retention of learning outcomes among participants, with 70.4% of course participants engaging in clinical trials as investigators, suggesting a long‐term impact on research involvement.

#### 3.1.5 EBM Curricula in Surgery

The development and implementation of EBM curricula in surgical education is evaluated in Tables 3 and 4. Across the studies, diverse methods, such as structured curricula or journal clubs, were employed to enhance EBM knowledge and critical appraisal skills among surgical trainees. While these approaches led to improvements in knowledge and engagement, they were often hindered by challenges like time constraints, limited attendance, and logistical issues. Surveys and program evaluations underscored gaps in EBM training among surgical trainees, with some programs being underutilized or lacking formal assessment and structured feedback. Overall, the findings suggest that although certain EBM training activities are beneficial, there is a need for better integration, accessibility, and structure to maximize their effectiveness in surgical education.

**TABLE 3:** Studies evaluating the development of EBM curricula in surgery.

Study	Method	Learners	Key findings
Santori et al. [25]	10‐session EBM program	Surgeons and health personnel	The 10‐session EBM program led to improved MCQ scores among participants, although attendance was limited due to clinical commitments. The program demonstrated effectiveness in enhancing EBM knowledge but faced challenges in engagement.
Haines and Nicholas [35]	Professor's rounds model	Neurosurgical trainees	The Professor's rounds model curriculum generated high self‐reported interest in promoting EBM among neurosurgical trainees. Participants were keen on integrating EBM principles into their surgical practice, indicating a positive reception to the curriculum.
Komenaka et al. [36]	Comprehensive evaluation of academic papers during breast surgery rotation	General surgery residents	During the breast surgery rotation, the comprehensive evaluation of academic papers significantly improved ABSITE scores and enhanced placement experience for general surgery residents. The curriculum was effective in improving EBM knowledge and satisfaction.
Temple and Ross [44]	Series of journal clubs with assigned homework	Plastic surgery residents	A series of journal clubs with assigned homework for plastic surgery residents showed a nonsignificant increase in knowledge levels post‐test, suggesting limited efficacy in teaching critical appraisal skills through this format.
Trickey et al. [45]	Structured curriculum with lectures, tutorials, and practice questions	General surgery residents	The structured curriculum, which included lectures, tutorials, and practice questions, resulted in significant improvement in post‐curriculum test scores for general surgery residents. This approach was effective in enhancing EBM knowledge and application.
Luc et al. [49, 50]	Debate‐style journal clubs	Cardiothoracic surgery trainees	Debate‐style journal clubs received mixed feedback from cardiothoracic surgery trainees. While the format improved engagement and exam performance, it was time‐consuming and faced logistical challenges. The approach was innovative but had practical limitations.
Lao, Puligandla, and Baird [57]	Quebec Pediatric Surgery Journal Club	Pediatric surgery trainees	The Quebec Pediatric Surgery Journal Club received high satisfaction scores from participants, with statistically significant improvement in knowledge and understanding of session content. However, 'knowledge decay' was observed due to the infrequent nature of the sessions.
Duong et al. [48]	Redesigned journal club series	Surgical residents	The redesigned journal club series, rotating between evidence‐based, deep‐dive, and landmark sessions, increased engagement, and improved critical appraisal skills among surgical residents. However, participants felt it was insufficient for staying updated with new information.

**TABLE 4:** Studies evaluating educational activity relating to EBM curricula in surgery.

Study	Method	Learners	Key findings
Siegrist and Giger [13]	Survey on curricular EBM training	Medical and surgical trainees	A survey on curricular EBM training revealed that surgical trainees reported almost no training in EBM, unlike their internal medicine counterparts, highlighting a significant gap in surgical education.
Ahmadi et al. [32]	EBRS program	General Surgery Residents	The EBRS program was useful for critical appraisal training among general surgeons, but only a small percentage of respondents utilized the full potential of the program. This suggests the need for better integration and utilization of EBM resources.
Williams et al. [51]	Survey on journal clubs	Surgical residents	Monthly journal clubs, as reported in the survey, were affected by time constraints, impacting attendance and effectiveness. Participants indicated that the format was not optimal for teaching statistics, pointing to the need for improvements.
Mullen and Sabri [53]	Survey on ophthalmology programmes	Ophthalmology residents	Survey on ophthalmology programmes identified journal clubs as crucial for clinical practice impact, emphasizing the importance of incorporating EBM knowledge into residency training to enhance clinical decision‐making.
Ibrahim et al. [56]	Survey on journal clubs in Nigeria	General, plastic, urological, and pediatric surgery residents	Survey on journal clubs in Nigeria highlighted the need for formal assessment of the quality of journal clubs. Residents expressed the need for structured feedback.

## 4 Discussion

This scoping review summarizes available literature evidence on the application of EBM in surgical practice. The studies, provide a detailed insight of the surgical community's knowledge and understanding, attitudes, availability of current opportunities, and tools and barriers relating to EBM.

Surgeons and surgical trainees value and understand elements of EBM as reflected in the studies evaluated. Most studies tried to quantify EBM knowledge by testing surgeons' awareness of EBM concepts. In none of the studies the included EBM concepts were derived systematically and validated, highlighting a need for a consensus curriculum for EBM within surgery. However, EBM concepts related to surgery [4, 67] are innumerable, and any consensus on the curriculum needs to highlight overarching concepts and specify minimum standards instead of a detailed syllabus.

Overall, surgeons and trainees were positive towards EBM. The studies highlight ingrained practices such as asking (colleagues) for advice instead of evaluating available evidence [14, 39]. Implementation of EBM curricula generally led to improvements in residents' skills and knowledge [35, 36, 45, 48, 49, 50, 57]. However, several barriers to the implementation of EBM into practice were highlighted, including time constraints [7, 14, 18, 19, 23, 24] and a lack of an appropriate educational infrastructure [7, 17, 20].

Furthermore, hierarchical structures [7, 18, 20] of traditional rank‐based systems within surgical teams may discourage open dialog or challenge to senior decision‐making, thereby limiting the integration of EBM into daily practice. Institutional barriers [7, 18, 20] encompass challenges such as limited funding, insufficient personnel, lack of leadership support for EBM initiatives, and inadequate access to training resources. Methodological inadequacies [16, 17, 20] reflect the difficulty in designing robust surgical studies due to the complexity of surgical interventions, variability in patient populations, and practical constraints in conducting randomized controlled trials in surgical settings. Access to evidence highlights disparities in the availability of up‐to‐date evidence‐based resources, such as journal access or EBM training, particularly in resource‐constrained environments.

The structure of EBM curricula varied among the different studies with all incorporating concepts relating to critical appraisal and biostatistics.

The scoping review identified a number of methods including journal clubs [55, 68], lectures [25], and courses or workshops [31, 40, 61] for the teaching and training in evidence‐based medicine. EBM can be assessed using various tools [9] that focus on different ‘steps of EBM’ [69]. The assessment tools utilized included the MacRae tool [46], the Berlin questionnaire [61], and the Fresno test [55] and some authors used nonvalidated tools [36, 68]. None of the available tools in the current literature focus on the ‘assess’ step of EBM, described in the consensus document on postgraduate EBM curricula [3].

Overall, education interventions do appear to improve knowledge; however this decreases over time, a phenomenon described as ’knowledge decay’ by Lao, Puligandla, and Baird [57]. It is not clear whether this decay was in part due to the interventions not considering system factors and being integrated appropriately into the wider curriculum. This would involve considering factors that impact on learning as illustrated in the 3P‐6Cs systems thinking toolkit [70].

It is important to note that surgical education stakeholders primarily in the USA and Canada, sought to establish an EBM curriculum, mainly because of educational requirements at national level (ABSITE score improvement, Canadian curriculum ’scholar’ indicators). Similar educational requirements are described in the Intercollegiate Surgical Curriculum Programme (ISCP) in the United Kingdom.

## 5 Limitations

The paucity of data in this field is a significant limitation of this review. Despite a systematic and comprehensive approach using an established framework [10], the studies retrieved were predominantly surveys, and heterogenous as to the population studied and the outcome measures included. Additional sources of heterogeneity included the differences in curricular structures, teaching and training methods. However, to our knowledge, this is the first study to review published evidence on evidence‐based practice in surgical specialties.

## 6 Conclusion

This scoping review highlights several areas for future work to advance the incorporation of EBM into surgical practice. Evidence suggests that effective strategies should not only address knowledge acquisition but also focus on practical application in clinical settings, aligning with the ‘shows how’ stage of Miller's pyramid [71]. Interventions such as case‐based learning, simulation, and decision‐making workshops may help bridge the gap between theoretical knowledge and clinical implementation. Additionally, addressing cultural and systemic barriers, including hierarchical structures, time constraints, and attitudes toward EBM, will be key to its broader acceptance and integration.

Future research should prioritize the development and evaluation of structured context‐specific EBM training programs and assessment tools for surgical trainees and trainers. Identifying ways to incorporate EBM into existing curricula without increasing the workload of surgical training is also critical. Furthermore, research into the role of leadership and institutional support in enabling EBM implementation could inform strategies to embed EBM as a routine part of surgical practice. These efforts can, in turn, nurture the proactive use of EBM in practice to become the norm.

## Author Contributions

**E. Tokidis:** conceptualization, data curation, writing – original draft, writing – review & editing. **G. Perin:** data curation, validation, writing – review & editing. **P. Vivekananda‐Schmidt:** validation, writing – review & editing. **S. P. Balasubramanian:** conceptualization, validation, writing – review & editing.

## Conflicts of Interest

The authors declare no conflicts of interest.

## Supporting information

Supporting Information S1

## Data Availability

The data used to produce this review are available upon reasonable request to the corresponding author.
