# Supplementary material for: Evidence‐Based Medicine Within Surgical Practice and Training: A Scoping Review
Source: World J Surg. 2025 Feb 4;49(4):916–29. doi: 10.1002/wjs.12479 (PMC11994140; doi:10.1002/wjs.12479)
Supplement: Supplementary file 1 — Supporting Information S1 [file WJS-49-916-s001.docx]

**Summary of database searches.**

**The White Rose Research Online search cannot be extracted as a table.**

| **PubMed** | **Strategy and results** |
| --- | --- |
| **Search Query** | ((Evidence-Based Medicine[MeSH Major Topic]) AND ((Surgery OR Surgical Practice))) AND ((Teach* OR assess* OR train* OR curricul* OR perception OR attitudes OR perception OR awareness OR utility OR need*)) |
| **All results** | 2127 |
| **Duplicates** | 15 |
| **Articles for title screening** | 2112 |
| **Excluded** | 1910 |
| **Included for abstact screening** | 202 |
| **Excluded** | 35 |
| **Included for full text screening** | 167 |
| **Excluded** | 138 |
| **Included for analysis** | 29 |

| **Database** | **Strategy** | **Citations** |
| --- | --- | --- |
| MEDLINE |  |  |
|  |  |  |
| 1 | evidence based surgery.mp. | 177 |
| 2 | (train* or teach* or perception or curricul* or assess* or awareness or attitude* or utility or need* or tool*).mp. | 8139437 |
| 3 | 1 and 2 | 89 |
| Comment 1 | Included articles after manual deduplication | 9 |

| **Database** | **Strategy** | **Citations** |
| --- | --- | --- |
| Embase |  |  |
|  |  |  |
| 1 | evidence based surgery.mp. | 209 |
| 2 | (train* or teach* or perception or curricul* or assess* or awareness or attitude* or utility or need* or tool*).mp. | 11693155 |
| 3 | 1 and 2 | 106 |
| 4 | limit 3 to "remove medline records" | 25 |
| Comment 1 | All articles excluded |  |
|  |  |  |

| **Database** | **Strategy** | **Citations** |
| --- | --- | --- |
| ERIC |  |  |
|  |  |  |
| 1 | exp evidence based practice/ | 5358 |
| 2 | exp surgery/ | 953 |
| 3 | (train* or teach* or perception or curricul* or assess* or awareness or attitude* or utility or need* or tool*).mp. [mp=abstract, title, heading word, identifiers] | 1365731 |
| 4 | 1 and 2 and 3 | 4 |
| Comment 1 | Search criteria modifed from other Ovid databases due to "evidence based surgery.mp" producing no results on ERIC |  |
| Comment 2 | All articles excluded from title screening due to irrelevance |  |
